# Shape-Stabilized Phase Change Materials for Solar Energy Storage: MgO and Mg(OH)_2_ Mixed with Polyethylene Glycol

**DOI:** 10.3390/nano9121773

**Published:** 2019-12-12

**Authors:** Md. Hasan Zahir, Mohammad Mizanur Rahman, Kashif Irshad, Mohammad Mominur Rahman

**Affiliations:** 1 Center of Research Excellence in Renewable Energy (CoRERE), King Fahd University of Petroleum and Minerals (KFUPM), Dhahran 31261, Saudi Arabia; kashif.irshad@kfupm.edu.sa; 2 Center of Research Excellence in Corrosion, King Fahd University of Petroleum and Minerals, Dhahran 31261, Saudi Arabia; mrahman@kfupm.edu.sa; 3 Department of Electrical Engineering, King Saud University, Riyadh 11451, Saudi Arabia; momin128@gmail.com

**Keywords:** MgO and Mg(OH)_2_ supporting materials, hydrothermal process, phase change material, blended or pure polyethylene glycol, solar thermal energy storage

## Abstract

Heat energy storage systems were fabricated with the impregnation method using MgO and Mg(OH)_2_ as supporting materials and polyethylene glycol (PEG-6000) as the functional phase. MgO and Mg(OH)_2_ were synthesized from the salt Mg(NO_3_)·6H_2_O by performing hydrothermal reactions with various precipitating agents. The precipitating agents were NaOH, KOH, NH_3_, NH_3_ with pamoic acid (PA), or (NH_4_)_2_CO_3_. The result shows that the selection of the precipitating agent has a significant impact on the crystallite structure, size, and shape of the final products. Of the precipitating agents tested, only NaOH and NH_3_ with PA produce single-phase Mg(OH)_2_ as the as-synthesized product. Pore size distribution analyses revealed that the surfaces of the as-synthesized MgO have a slit-like pore structure with a broad-type pore size distribution, whereas the as-synthesized Mg(OH)_2_ has a mesoporous structure with a narrow pore size distribution. This structure enhances the latent heat of the phase change material (PCM) as well as super cooling mitigation. The PEG/Mg(OH)_2_ PCM also exhibits reproducible behavior over a large number of thermal cycles. Both MgO and Mg(OH)_2_ matrices prevent the leakage of liquid PEG during the phase transition in phase change materials (PCMs). However, MgO/PEG has a low impregnation ratio and efficiency, with a low thermal storage capability. This is due to the large pore diameter, which does not allow MgO to retain a larger amount of PEG. The latent heat values of PEG-1000/PEG-6000 blends with MgO and Mg(OH)_2_ were also determined with a view to extending the application of the PCMs to energy storage over wider temperature ranges.

## 1. Introduction

The demand for renewable energy is increasing very rapidly because non-renewable resources are steadily being exhausted, so the development of sustainable renewable energy sources has attracted world-wide attention. In this respect, the harvesting of solar energy is dominant. However, the times at which solar energy is available and the periods of high energy utilization are different. Therefore, the storage of solar energy is essential so that it can be released in times of demand [1]. A sophisticated energy storage system could also be utilized in waste heat recovery, where similar issues arise [2]. Thermal energy storage systems can be utilized in hot and cold climates and their availability would resolve the time mismatch between energy supply and demand.

Sensible and latent heat storage are the two main approaches used to store thermal energy. Sensible heat storage have advantages such as high heat storage capacity but also possesses certain drawbacks as efficiency of storage depends upon temperature of storage material, which is difficult to maintain at a constant temperature under real scale operations [3,4]. On the other hand, latent heat storage is in high demand because of its high energy storage density and the ability to store heat at a constant temperature, thus latent heat storage phase change materials (PCMs) have been extensively studied over the last decade.

Among the organic PCMs, polyethylene glycol (PEG) is a very good candidate due to its highly desirable features. Phase transition temperature and high heat storage capacity [5] of PEG, which can be controlled by changing its molecular weight and/or mass fraction, are very favorable for PCM applications. Exceptional resistance to corrosion, good chemical and thermal stability, non-toxicity, low vapor pressure, and low cost [6] are some of the other unique properties of PEG. However, PEG also has some shortcomings, including very low thermal conductivity and leakage in time of phase transition. Attempts have been made to solve these problems by encapsulating PEG with a metal or an alloy. But encapsulation itself leads to supercooling, and it is a laborious, complicated, and time-consuming process [7]. The use of shape-stabilized or form stable composite phase change materials (ss-CPCMs), which can be prepared by mixing PEG with carrier matrices, is an innovative method employed to overcome the above problems [8].

The advantages of this technology are (i) the organic functional phase can maintain its activity without creating the leakage problem even when the phase-change substance changes from solid to liquid state [9,10]; and (ii) the carrier matrix can accommodate and/or the liquid PCMs can penetrate into the pores of the matrix. As a result, the functional organic phase can be active inside the porous matrix, which can help protect the organic PCM from destructive connections with the nearby materials and environment during real application [11,12]. In fact, liquid PCMs are not suitable for real-world applications, such as the use as building materials.

As a matrix, silica, fatty acid, diatomite, expanded perlite, and graphite have been studied extensively. In addition, metal foam, vermiculite, bentonite, and montmorillonite are also well-known [13,14,15,16]. Several research groups have evaluated the use of a polymer as a matrix. However, as encapsulation is essential when a polymer is used, the cost of fabrication of the PCM is increased. In this case, the incompatibility is an issue, which needs to be solved. Usually, the heat resistance could be enhanced by the shell of the organic capsule. Moreover, it is known that the organic shells are unable to stop the leakage of PCMs [17]. Hence, selecting an appropriate inorganic carrier matrix for ss-CPCMs can be a promising idea, which may lead to sustainable PCM technology.

Inorganic porous materials have attractive features, such as excellent chemical and thermal stability, unique sorption properties, high specific surface area, a porous structure, high thermal conductivity, high fire-retardancy, and a simple preparation method [18]. As organic materials do not possess most of these properties, inorganic porous materials are more appealing to be used as matrices for phase change materials (PCMs). Inorganic matrices may also help in preventing liquid leakage and enhance thermal conductivity. Py et al. have shown that a mesoporous structure performs better as a matrix compared to a micro or macroporous support [19]. However, Py et al. do not provide any isotherms for the matrix materials with a mesoporous structure they studied. In fact, Py et al. arrived at the conclusion based on the properties of paraffin waxes supported within the porous structure of the inorganic silica matrix materials and activated carbon, citing references 18, 19, and 20 of [19]. However, reference 18 of [19] is about modeling and experimental studies of phase-change heat regenerators, and not silica catalysts. Reference 19 of [19] is in French, and reference 20 of [19] is on the dynamics of latent heat storage in fixed beds, employing a non-linear equilibrium model and an analogy with chromatography. The results in references 18, 19, and 20 of [19] do not appear to support the conclusions drawn by Py et al. In fact, data on pore size distribution, pore volume, and pore diameter are not available in the cited references, and the effects or impact of these properties on PCMs have not been convincingly demonstrated. All conclusions of Py et al. are based on modeling studies, and they used a mathematical equation to calculate the total porosity.

Feng et al. have also studied the phase change behavior of ss-CPCMs, prepared with PEG and porous materials (i.e., AC (activated carbon) and silica templates (MCM-41, SBA-15)). They found that the molecular weight of PEG and the pore structure of porous support significantly affect the enthalpy of these composites [20]. However, they have not provided any experimentally determined pore size distribution isotherms for the porous support. Shudong et al. have demonstrated that supercooling can be minimized significantly by fabricating ss-CPCMs with porous matrices [21]. They have also not provided any experimentally determined pore size distribution isotherms. 

Finally, the published results indicate that the porous structure can play a significant role when the crystallization and phase change are influenced by the capillary forces, the surface tension of meso-, micro-, or macro-pores, and the surface area of the supports. Recently, Tingting et al. [22] have found that a mixed meso- and macro-porous support is better for retaining liquid polymers, resulting in a high latent heat. Ho et al. have provided nitrogen adsorption–desorption isotherms for the MgO support. However, the system they used has a low melting enthalpy of 61.62 J/g, probably due to the large pore diameter of the support [23]. In fact, the energy-storage efficiency of all above-mentioned ss-CPCMs depends on the porous structure of the supporting materials. The crystallization environment, especially if it is mesoporous or nano-porous, alters the crystallization behavior and the phase transition process. Thus, the analysis of the relationship between the matrix and the heat storage ability, the crystallization behavior, and the thermal stability of PCMs is an important area of research. The fact that the porous structure (i.e., the pore size distribution and geometric structure) can influence the crystalline behavior in supports with small pores is well established. Thus, in supports with small pores the molecular motion of PCM can be hindered, resulting in a decrease of the latent heat storage capacity [22,24,25]. On the other hand, materials with macro pores cannot exert an adequate capillary force to retain the PCM within the matrix when it melts. Mesoporous materials show the best performance as supports. However, recent studies indicate that microporous Zeolite Socony Mobil–5 (ZSM-5) [25] as well as AC [26] are ideal supporting materials for overcoming defects of PCMs containing PEG. The thermal reliability of the PEG/ZSM-5 composites is also higher during the phase change cycles of melting [25]. Even though microporous supporting material, such as SiO_2_ [27,28], ZSM-5 [25], and AC [26], decrease the latent heat value, they increase the conductivity as well as the thermal stability of the PCM. Porous material has been used extensively in the field of catalysis, sorption, chemical engineering, energy, environmental protection, and in reaction chambers. However, inorganic porous materials have not been explored extensively in the field of phase change technology for thermal energy storage. In fact, the mechanism of energy storage in ss-CPCM and its relationship with the porous support materials are not clearly understood or reported in the open literature. There are two distinctive features of the Type H3 loop of mesoporous materials. One is the common mesoporous materials [23,29] and the other is the porous network containing meso- and macro-pores. In addition, most of the published literature did not provide the pore volume of their support, which is highly important for PCM accommodation. The accommodated PCM in the porous support can play a vital role in the high latent heat value of the ss-CPCM system 

In this study, MgO and Mg(OH)_2_ were chosen as support materials because MgO has a higher thermal conductivity (~48.4 W m^−1^·K^−1^) than other oxide materials [30]. MgO nanoparticles have been prepared with various methods such as pulse laser deposition [31], laser ablation [32], and the thermal decomposition of hydroxides or carbonates [33]. However, these methods usually produce nanoparticles with relatively large and varied grain sizes, inhomogeneous morphologies, and small surface areas. Cui et al. reported the synthesis of mesoporous MgO with a template-free hydrothermal method, but this approach required the calcination of the precursor in air at 600 °C for 2 h [34]. Although the characteristics of inorganic materials, particularly those of the alkaline and alkaline earth metal nitrates, are promising and advantageous for their use in PCMs, some problems must be overcome for these materials to be used in commercial products. Only the alkaline salts and salt hydrates of Li, Na, K, Mg, Ca, and Ba have been tested as PCM materials; very few studies of PCMs based on alkaline hydroxide powders have been performed [35]. The Mg(OH)_2_ possess many advantages and can be used as an additive in fertilizer, flame retardant with non-toxic nature, neutralizer for acidic contaminants for both liquid and gases, filler for the paper industry, and antibacterial agents [36]. Further this novel PCM can be used in the construction industry, especially for hot climatic regions, and for waste heat recovery and solar energy applications depending on temperature range requirements [25].

Recently, Sulaiman et al. [37] reported the preparation of In-Sn-O nanoparticles with the hydrothermal method by using the disodium salt of PA as an organic additive. PA has been utilized as a stabilizer, a surfactant, a reductant, and even as a ligand; its use in their study enabled the control of the sizes and shapes of the nanoparticles. PA acts as a capping and reducing agent that stabilizes nano-intermediate products. In this study, porous MgO and Mg(OH)_2_ were prepared with the hydrothermal method at low temperatures in the presence or absence of PA. The effects of the use of various types of precipitating agents were examined. The synthesized phase-pure porous MgO and Mg(OH)_2_ were mixed with PEG or a PEG blend in order to fabricate novel ss-PCMs. The latent heat and thermal properties of the PCMs were examined with various analytical techniques to assess their potential in real applications.

## 2. Methodology

### 2.1. Chemicals

PEG 6000 and PEG 1000 were purchased from the USB Corporation, Cleveland, OH, USA. Mg(NO_3_)_2_·6H_2_O and ethyl alcohol, Pamoic acid, and NH_3_ were purchased from Sigma-Aldrich, St. Louis, MO, USA.

### 2.2. Hydrothermal Synthesis

The weight of 6.00 g Mg(NO_3_)_2_·6H_2_O was used for the preparation of either MgO or Mg(OH)_2_. First of all, 6.00 g Mg(NO_3_)_2_·6H_2_O was dissolved in 100 ml deionized water. The pamoic acid of 0.20 g was also dissolved in 10 mL deionized water and added into the above Mg(NO_3_)_2_·6H_2_O solution. Then an appropriate amount of NH_3_ (4.1 mol cm^−3^) was added to the solution for co-precipitation and pH of the solution was adjusted 9.00. The whole solution was well-stirred during the pH adjustment using NH_3_. The precipitate was transferred to a Teflon container and the container was put into a steel vessel. The container mouth was closed very tightly and then the container was put in a furnace for hydrothermal reaction. The hydrothermal reaction was operated at 200 °C for 24 h. To discard the impurities, the resulting product was firstly washed with deionized water and then with ethanol using centrifuging process and eventually dried at 120 °C in an oven. The same procedures were followed for preparing other samples. The precipitate of these samples are prepared without PA and two other samples were prepared by adding NaOH and KOH for the purpose of comparison.

### 2.3. Preparation of the Shape-Stabilized Composite PCM

By mixing 0.5 g of PEG (6000 or 1000) and MgO or Mg(OH)_2_ 0.2 g in 50 mL of absolute ethanol, PCM composite structure was prepared. The solution was then sonicated for half an hour. Ethanol was evaporated at 80 °C while the solution was kept stirring. Finally, composite of PCMs from PEG/MgO and PEG/Mg(OH)_2_ were acquired and further characterized by various techniques.

### 2.4. Characterization

The X-ray diffraction (XRD) patterns were recorded using a powder X-ray diffractometer (Rigaku, Tokyo, Japan) with Cu-Kα radiation, operated at 30 kV and 15 mA. FT-IR spectroscopy (Impact 400D, Nicolet, Madison, WI, USA) was used to characterize the PCMs. The field emission scanning electron microscope (FE-SEM) images were obtained using a JEOL JSM-6400F (JEOL USA Inc., Peabody, MA, USA) at an acceleration voltage of 10 kV. Energy-dispersive X-ray spectra (EDS) were recorded with an Xmass detector (JEOL USA Inc., Peabody, MA, USA). TEM images were obtained using a transmission electron microscope (JEOL, JEM 2011, JEOL USA Inc., Peabody, MA, USA) with a 94 k CCD camera operated at 200 kV. Using a NOVA-1200 apparatus (JEOL USA Inc., Peabody, MA, USA), the specific surface area, pore volume, and pore diameter of the samples were determined. BET surface area measurements were carried out with a Tristar II 3020 system. The powders were evacuated for 3 h at 200 °C, and the N_2_ adsorption isotherms of the catalysts were obtained in liquid N_2_ (−196 °C). The pore size distributions were obtained with the Barrett–Joyner–Halenda (BJH) formula. The chemical composition of the samples was investigated by X-ray photoelectron spectroscopy (XPS) using an X-ray photoelectron spectrometer (ESCALAB-250, Thermo-VG Scientific, Waltham, MA, USA), with Al-Kα radiation (1486.6 eV). XPS spectra were taken in a specimen chamber at ambient temperature and a pressure of 5 × 10^−10^ m bar. Thermal gravimetric analysis was performed in a Pyris 6 TGA (Perkin Elmer, Shelton, CT, USA). The heating rate was maintained at 10 °C/min from room temperature to 600 °C under dry nitrogen. A differential scanning calorimeter (DSC, Q2000, TA, New Castle, DE, USA) was used for the phase change temperature and the latent heat of the samples. The DSC measurements were conducted by heating 10 mg samples sealed in an aluminum pan at a heating rate of 5 °C/min under a constant stream of argon at a flow rate of 20 mL/min. The thermal conductivity of the casted films was measured using TCi Thermal Conductivity Analyzer (C-Therm Technologies, Fredericton, Canada, which uses the modified transient plane source method (MTPS) for the measurement.

## 3. Results and Discussion

### 3.1. Characteristics of the Synthesized Products

#### 3.1.1. XRD Analysis

**Figure 1:**
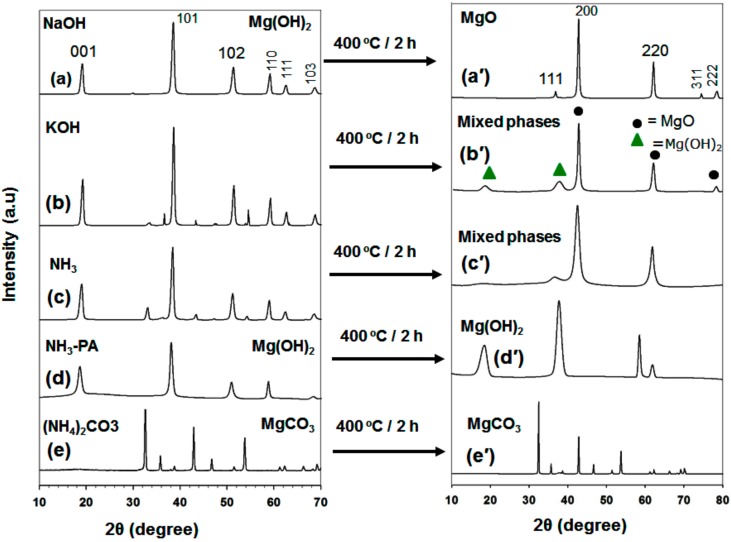
XRD pattern of as-synthesized powders prepared by (**a**) NaOH, (**b**) KOH, (**c**) NH_3_, (**d**) NH_3_-PA, and (**e**) (NH_4_)_2_CO_3_ precipitating agents, and the corresponding (**a**′), (**b**′), (**c**′), (**d**′), and (**e**′) are the calcined samples at 400 °C for 2 h.

Initially, an alkaline solution like NaOH and KOH were tested as precipitating agents of MgO and Mg(OH)_2_ from its nitrate salt. As precipitating agents NH_3_, NH_3_ with pamoic acid, and (NH_4_)_2_CO_3_ were also tested for the same purposes (Figure 1a–e).

Figure 1a shows that when NaOH was used as the precipitating agent, Mg(OH)_2_ as-synthesized powder was obtained, which could be converted into phase-pure MgO through calcination at 400 °C for 2 h in air (Figure 1a, right, top). All the diffraction peaks in Figure 1a’ correspond to those of cubic MgO (i.e., those in JCPDS card number 45-0946 for space group *Fm*-3m (225)). These very intense and clear peaks indicate that the product has good crystallinity. No peaks due to impurities are evident. In the case of KOH, the as-synthesized product produces a mixture of Mg(OH)_2_ and MgO diffraction peaks. When NH_3_ alone is used as the precipitating agent, mixed phases result. In contrast, phase-pure Mg(OH)_2_ is formed after the addition of NH_3_ and pamoic acid (Figure 1d). It is surprising that the positions of the peaks in the XRD pattern of Mg(OH)_2_ prepared by the addition of NH_3_ and PA do not change (i.e., its phase structure is unaltered), after calcination at 400 °C for 2 h. All the peaks are slightly broader after heat treatment, which indicates that this Mg(OH)_2_ product has a small grain size. It is likely that the PA acts in this case as a capping agent that stabilizes the nanoparticles. The as-synthesized powders arising from the addition of (NH_4_)_2_CO_3_ consist of a pure MgCO_3_ phase. In all cases, the desired MgO porous support was not obtained from hydrothermal synthesis alone. As discussed above, a calcination step is mandatory in the synthesis of phase-pure MgO with hydrothermal or other processes. Recently, Hao et al. synthesized MgO with a complicated and time-consuming synthesis process that involved the use of Pluronic F127 co-polymer and calcination at a high temperature to produce a porous structure [23]. The XRD pattern of as-synthesized Mg(OH)_2_ powders (Figure 1a) had mixed phases after heat treatment at 250 °C for 2 h. The Mg(OH)_2_ powders (Figure 1a) was obtained by precipitating agent NaOH. Moreover, the shape of as-prepared Mg(OH)_2_ powders had sheet-like structure with very low surface area. On the other hand, Mg(OH)_2_ prepared by NH_3_-PA precipitating agent had a high surface area with flower-type morphology (Figure 1d′). The MgO and Mg(OH)_2_ samples discussed in the following section are those for which the X-ray diffraction patterns are shown in Figure 1a′,d′, respectively, unless otherwise noted.

**Figure 2:**
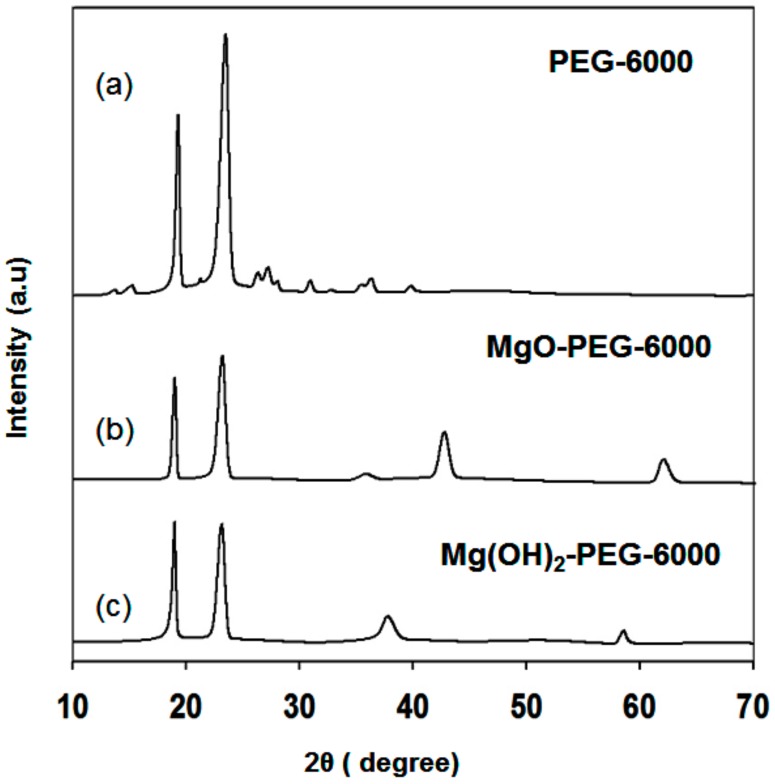
XRD pattern of (**a**) pure PEG-6000, (**b**) PEG/MgO, and (**c**) PEG/Mg(OH)_2_ composites.

The X-ray diffraction patterns of (a) PEG-6000 (PEG) alone and the composites (b) PEG/MgO and (c) PEG/Mg(OH)_2_ PCM are shown in Figure 2. The X-ray intensities of PEG alone are higher than those of MgO and Mg(OH)_2_ composites. It seems that the pores of MgO and Mg(OH)_2_ were occupied by the melted PEG. As a result, a decrease in the crystallite size of PEG has occurred in the composites. The X-ray diffraction pattern of PEG/MgO had mixed phases due to the presence of MgO and PEG peaks. Only PEG and Mg(OH)_2_ peaks were observed in the case of PEG/Mg(OH)_2_ PCM composite sample. It indicates that the PEG/MgO and PEG/Mg(OH)_2_ composites are fully mixed. It means no chemical interaction has occurred between PEG and MgO or Mg(OH)_2_ because no new peak was observed.

#### 3.1.2. FTIR Analysis

**Figure 3:**
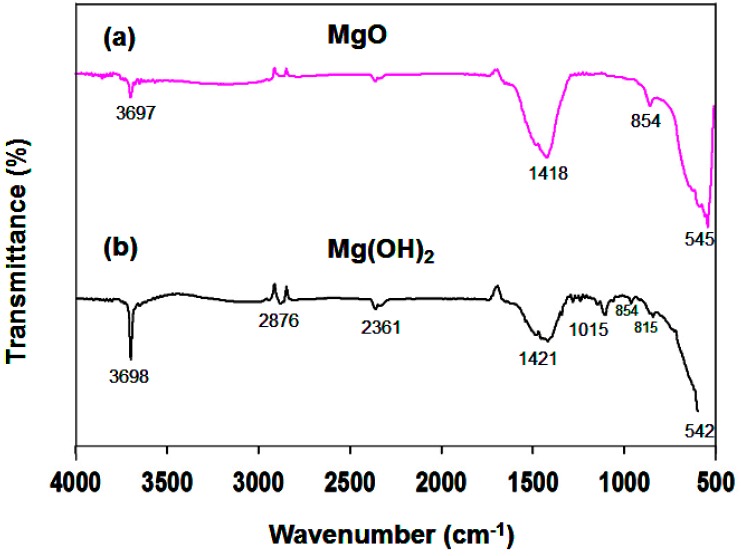
FTIR spectra of (**a**) MgO, and (**b**) Mg(OH)_2_.

Figure 3 shows the FTIR spectra of (a) MgO and (b) a calcined Mg(OH)_2_ sample prepared in the presence of NH_3_ and PA. The band near 3697 cm^−1^ is attributed to the stretching of H–O–H. It is well known that MgO surfaces readily absorb H_2_O and CO_2_ molecules when exposed to the atmosphere. The peaks at 1418 cm^−1^ are assigned to the asymmetrical and symmetrical stretching vibrations of carboxylate (O–C=O), which could be due to the presence of minor impurities in the precursors used during the synthesis process. The absorption band near 854 cm^−1^ is characteristic of cubic MgO [38]. The characteristic vibrational frequency at 545 cm^−1^ is in good agreement with values reported elsewhere. Figure 3b contains a sharp and intense peak at 3698 cm^−1^, which is due to the –OH group in Mg(OH)_2_; its intensity is higher than that of the band near 3697 cm^−1^ in the MgO spectrum.

**Figure 4:**
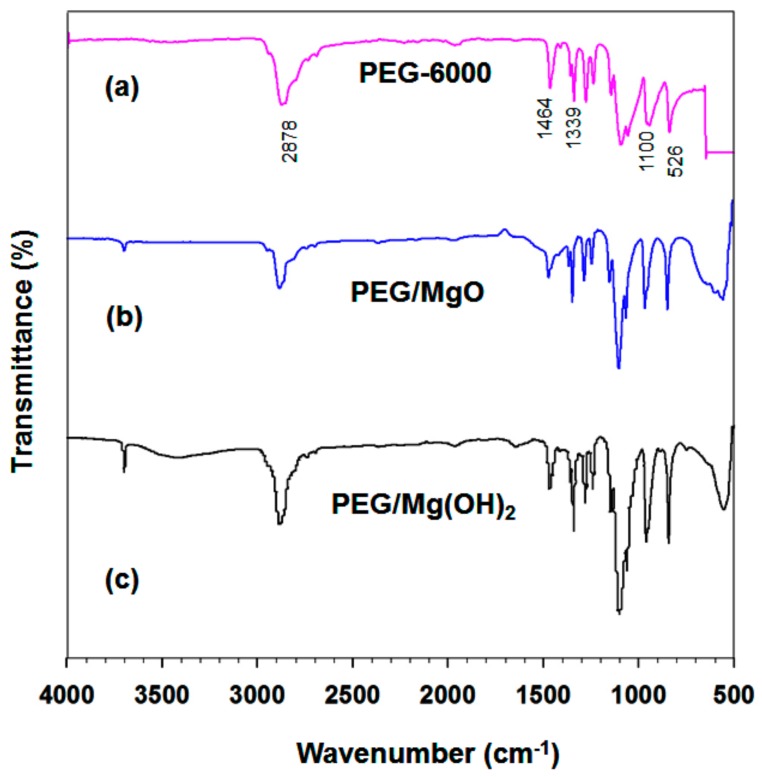
FTIR spectra of (**a**) pure PEG-6000, (**b**) PEG/MgO, and (**c**) PEG/Mg(OH)_2_ composites.

The interactions between PEG and the supporting materials were characterized by analyzing the samples with FTIR spectroscopy at room temperature. Figure 4 shows the FT-IR spectra of (a) PEG, (b) MgO/PEG, and (c) a calcined Mg(OH)_2_/PEG sample. An absorption band at 3698 cm^−1^ due to the stretching of OH groups is evident for all samples except PEG. For the PEG sample, the 2878 cm^−1^ peak is due to aliphatic C–H stretching. The peaks at 1464 and 1339 cm^−1^ are due to C–H bending vibrations. The O–H and C–O–H stretching vibrations produce peaks at 1278 and 1095 cm^−1^ respectively; similar results were obtained in a previous report.

In the PEG spectrum, the peak at 1100 cm^−1^ is due to the stretching vibration of C–O–C. The strong peaks at 2878 and 962 cm^−1^ result from the stretching vibrations of the functional group-CH_2_ [39]. Figure 4b shows the MgO/PEG spectrum, which contains peaks due to both MgO and PEG. No significant new peak is evident; it seems that MgO and PEG are well mixed, with only physical mixing occurring. The FTIR spectrum of PEG/Mg(OH)_2_ shown in Figure 4c contains similar characteristic peaks due to Mg(OH)_2_ and PEG. There are no additional peaks and no shifts of the individual peaks in the spectrum of the PEG/Mg(OH)_2_ composite. Therefore, we conclude that the combination of Mg(OH)_2_ and PEG results only in the physical absorption of PEG into the Mg(OH)_2_ matrix and no chemical interactions arise.

#### 3.1.3. Microstructures

**Figure 5:**
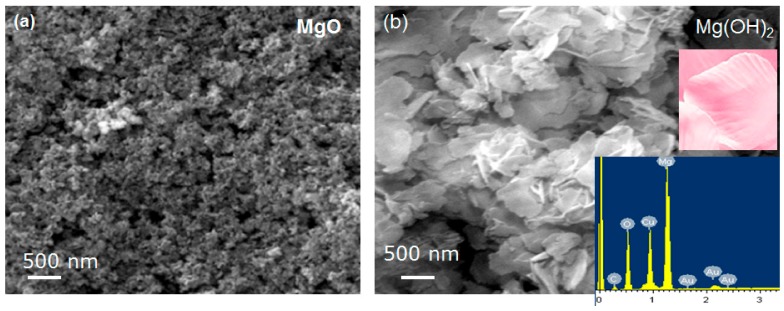
The field emission scanning electron microscope (FE-SEM) images of (**a**) MgO and (**b**) Mg(OH)_2_ composites (inset in Figure (**b**), is a digital photograph of a real rose flower petal (top) and EDX spectrum of Mg(OH)_2_ (bottom).

The morphologies and microstructures of the MgO and Mg(OH)_2_ samples after heat treatment at 400 °C for 2 h were examined by performing scanning electron microscopy, and the results are shown in Figure 5.

As is typical, the heat treatment results in the agglomeration of the powder due to the interaction between the nanoparticles. The surfaces of the MgO nanoparticles were analyzed and found to exhibit considerable surface roughness. It was noted that many pores of macro size and voids are evident in these SEM images (Figure 5a). The Mg(OH)_2_ powders in Figure 5b consist mainly of thin flakes. These flakes look like the nanopetals of rose flowers composed of thinner nanoplates. All the petals have similar shapes and sizes; a real petal can be seen in the inset in Figure 5b. The EDX spectrum in the inset of Figure 5b shows that the Mg(OH)_2_ sample contains only Mg, O, and gold (Au), which is present in the coating used in preparation for SEM. The SEM mapping indicates that all these elements are homogeneously distributed.

**Figure 6:**
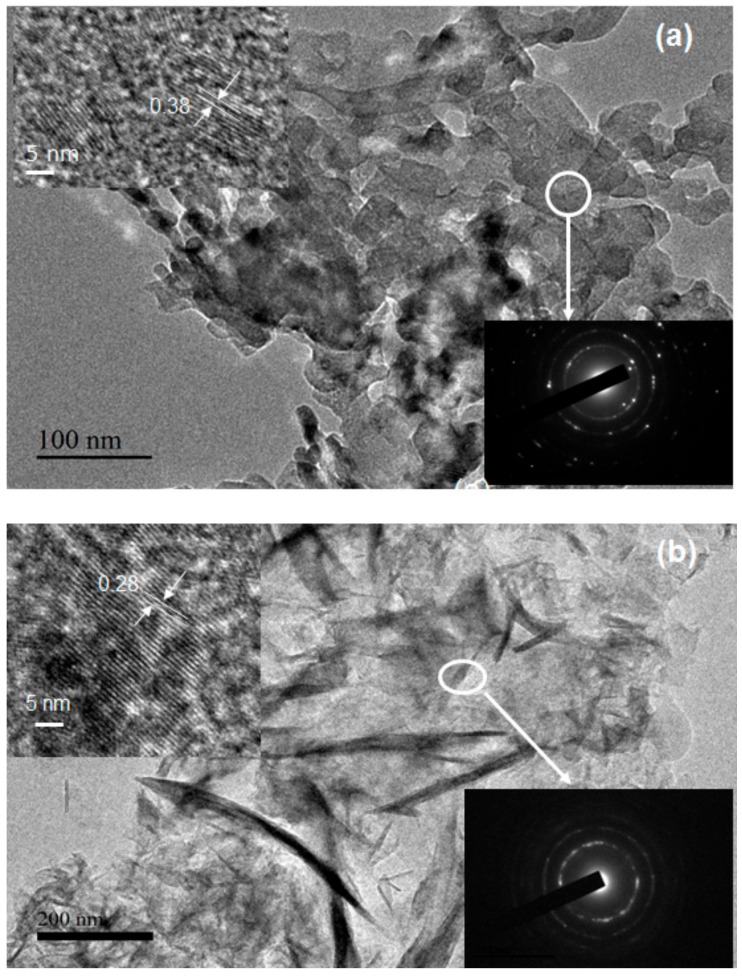
TEM images of (**a**) MgO and (**b**) Mg(OH)_2_ composites and the corresponding high-resolution transmission electron microscopy (HRTEM) image with the SAED image in the inset.

Figure 6 shows transmission electron microscopy (TEM) and high-resolution TEM (HRTEM) images of (a) MgO and (b) Mg(OH)_2_. The TEM images show that the MgO powders consist of agglomerated sheets. The selected area electron diffraction (SAED) pattern was obtained from the area on the MgO surface marked by the white circle in Figure 6a; the interplanar spacing in the MgO sample is 0.366 nm. Furthermore, the SAED pattern (see the inset in Figure 6a) comprises discontinuous rings, which indicates that the sample consists of polycrystals.

Figure 6b shows a TEM image of Mg(OH)_2_. All these particles appears to be amorphous with thin films type morphology. Ultrafine particles with large specific surface areas usually readily agglomerate to form larger particles. In this case, PA probably reduces the free energy and prevents agglomeration, which restrains the growth of Mg(OH)_2_. The chemical structure of PA could facilitate the formation of particles with fibrous texture under hydrothermal conditions and subsequent heat treatment. The SAED pattern (see the inset in Figure 6b) obtained from the fibers indicates that they are crystalline. The spacing between the lattice fringes was found to be 0.280 ± 0.004 nm (Figure 6b). The SAED pattern (Figure 6b inset) contains rings that are not fully continuous, which suggests that the Mg(OH)_2_ powders are less crystalline than the MgO powders.

#### 3.1.4. XPS Analysis

**Figure 7:**
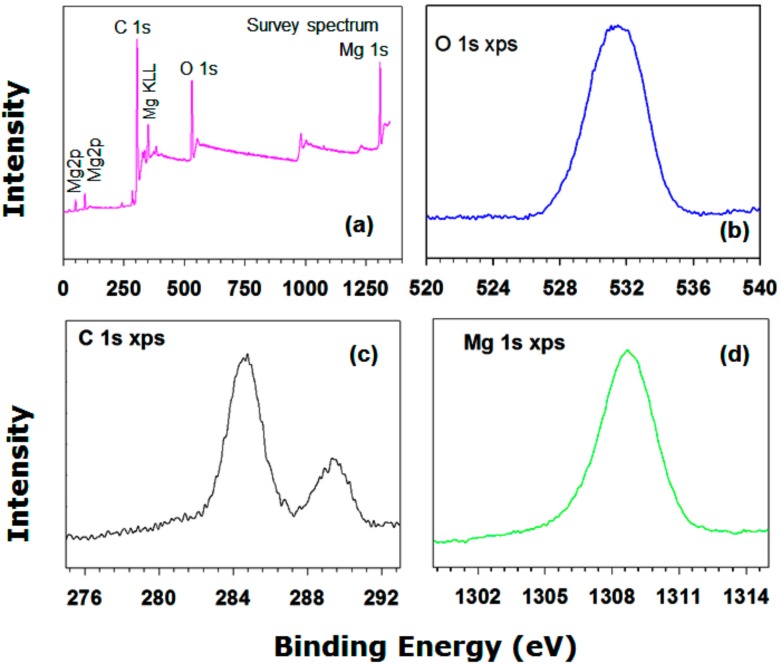
XPS spectra of Mg(OH)_2_: (**a**) Survey spectrum, (**b**) O 1s region, (**c**) C 1s region, (**d**) Mg 1s region,

Figure 7 shows the XP spectrum of the Mg(OH)_2_ powders, which provides the surface composition of the sample. Characteristic peaks for elemental magnesium, oxygen, and carbon are clearly evident. At high resolution, one Mg 1s XPS peak can be distinguished at 1307 eV. Two peaks due to carbon species are evident in the C 1s spectrum (Figure 7c). The C_II_-type peak is due to carbon–oxygen bonds. At 532.5 eV, a broad O 1s XPS peak is evident for the as-prepared sample. The Mg 1s core level spectrum can be resolved into three component peaks. The high binding energy component peak is due to Mg(OH)_2_. It is worthwhile to mention that the presence of OH group on the surface of PCM has potential impact on minimizing the supercooling effect [4]. The other two-component peaks are attributed to Mg and magnesium oxide (results not shown). The O 1s high binding energy component is attributed to oxygen species (such as water) adsorbed onto Mg, which results from the exposure of the sample to air and the hydrothermal process.

#### 3.1.5. Effect of Pore Size Distribution and Pore Volume

**Figure 8:**
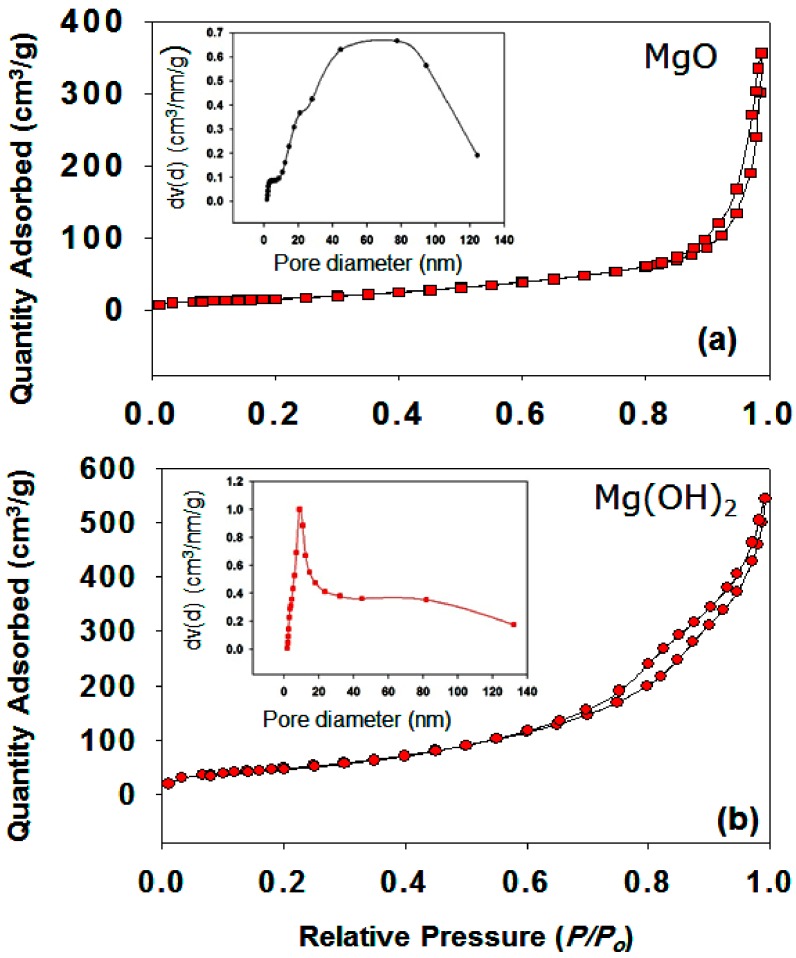
Nitrogen adsorption–desorption isotherms of (**a**) MgO and (**b**) Mg(OH)_2_ samples. The inset of the figure shows the pore diameter for the same samples.

A porous materials can be applied in many fields; however, it depends on their porosity, pore size, pore size distribution, pore shape, pore morphology, and the specific surface area. In fact, porosity has a high influence over the mechanical, physical, and chemical properties of the materials. The nitrogen adsorption–desorption isotherms of MgO and Mg(OH)_2_ powders are shown in Figure 8a,b. The pore size distribution curves for the same samples can be seen in Figure 8a,b (please see insets).

A sharp N_2_ adsorption–desorption peak of MgO was observed in the high *P/P*_0_ range, which indicates that the materials contain both large mesopores and macropores (Figure 8a). As mentioned above, a matrix with very small pores alters the crystalline behavior and the material may not be able to relax to its lowest energy state. Hence, a low latent heat enthalpy value will be obtained [17]. Very large pores are also not suitable as they cannot confine the melted PCM within the matrix. Figure 8a (inset) shows that the MgO has large pores in the range of 20 to 120 nm, possibly as a result of etching of MgO particles.

Figure 8b shows an IV type isotherm for the Mg(OH)_2_ sample with an apparent H4-type hysteresis loop in the *P/P*_0_ range of 0.6 to 1, clearly indicating the presence of a mesoporous structure. The mesoporous structure is an excellent support material for ss-CPCMs [17,19,21]. The surface areas of MgO and Mg(OH)_2_ were determined to be 63 and 184 m^2^g^−1^, respectively. The pore size distributions of Mg(OH)_2_ indicate micro size pores (e. g., below 20 nm (see the inset in Figure 8b)). The mesoporous structures strongly favor the absorption of PEG by capillary force, which enhances the thermal dependability of the PEG/Mg(OH)_2_ PCM during melting and freezing cycles. Figure 8b (inset) shows that the Mg(OH)_2_ sample had a very low pore volume, probably due to the incorporation of PA species into the stacking pores of Mg(OH)_2_. On the other hand, the MgO sample had a broader pore size distribution and lower pore volume than the Mg(OH)_2_ sample (Table 1). Moreover, very poor performance was observed in the case of MgO, probably due to the presence of meso- as well as microporous pore size distribution. Phenomena dependent on the porous structure were observed experimentally in the case of MgO and Mg(OH)_2_ in Table 1.

#### 3.1.6. Thermal Stability of the Composites

**Figure 9:**
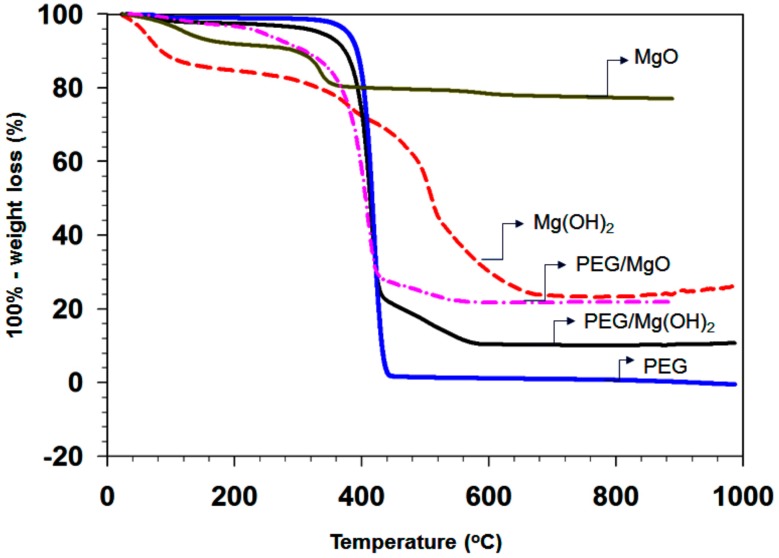
TGA curves of MgO, Mg(OH)_2_, PEG/MgO, PEG/Mg(OH)_2_, and pure PEG samples, all samples are marked by arrow sign.

The TGA analyses of the hydrothermally treated powders were carried out in a N_2_ flow at a heating rate of 5 °C /min. The TGA curves for pure PEG (blue), MgO (gray), as-prepared Mg(OH)_2_ (red), and the PEG/Mg(OH)_2_ (black) composite are shown in Figure 9.

The initial weight loss of the MgO sample is 12% up to 200 °C due to the elimination of absorbed water and hydroxides group. In the second step, approximately 8% weight loss arises in the temperature range 370 to 800 °C, due to the elimination of chemically adsorbed H_2_O and oxidation of remaining organic compounds [40]. Mg(OH)_2_ starts to lose weight just after the heating is commenced; around 70% is lost up to 650 °C. There are two main zones in the weight loss of Mg(OH)_2_. As shown in Figure 9, the as-synthesized PEG/MgO and PEG/Mg(OH)_2_ PCMs show very good thermal stability below 380 °C, which is much higher than the phase transition temperature of PEG. The weight loss curves of all samples contain only one step. The thermal stability of the PCM is another important index for real heat storage applications. The addition of PEG can enhance the hydrophobic properties of Mg(OH)_2_, which probably improves its stability and extends its application possibilities as an active filler [41]. For the PEG/Mg(OH)_2_ PCMs sample, no mass loss was observed below ca. 420 °C, indicating thermal stability to this point.

In a nitrogen atmosphere at 500 °C, the weight loss of PEG-6000 is 95.97%, which corresponds to the pyrolysis of the PEG functional groups. Pure PEG-6000 began to melt at about 78 °C. The abrupt melting was started at 400 °C and the total weight loss percentage was 100% at around 440 °C. The TGA analysis shows that the as-synthesized PEG/Mg(OH)_2_ powders decompose into CO_2_ and MgO near 400 °C. The weight loss of the porous PEG/Mg(OH)_2_ is almost 100% during heating up to 420 °C due to the removal of the absorbed water and hydroxyl groups. Note that the PEG/Mg(OH)_2_ sample has a higher thermal stability than PEG/MgO. The weight loss of Mg(OH)_2_ commences at around 10 °C and that of MgO near 100 °C, whereas the weight loss of PEG/Mg(OH)_2_ starts from 220 °C. Thus PEG is homogeneously dispersed into the porous Mg(OH)_2_ support and strongly attached to the pore wall. The total weight loss percentage arising for the PEG/Mg(OH)_2_ composite is 78.2% upon heating up to 500 °C. The composites contain 28.6% (0.2 g) of the matrix and 71.4% (0.5 g) of PEG. The total weight of the MgO/PEG and Mg(OH)_2_/PEG composites at 500 °C is 24.21% and 12.12% higher than that of PEG, respectively.

These results suggest that the porous Mg(OH)_2_ support increases the thermal stability of PEG, probably by creating a defensive barrier. Hu et al. have reported that ethanol and PEG-6000 can work effectively as dispersing agents and reduce the average size of Mg(OH)_2_ samples by preventing the agglomeration of particles [42]. Thus, most likely PEG is highly dispersed inside the mesopores in the case of PEG/Mg(OH)_2_, while in the case of PEG/MgO it is not. Hu et al. also claim that PEG-6000 has no significant influence on the crystal structure, morphology, and size uniformity of MgO nanoparticles [42,43].

#### 3.1.7. DSC Results of the PEG/MgO and PEG/Mg(OH)_2_ Composites

PEG has a melting temperature range that can be tuned from 32 to 60.7 °C by varying its molecular weight. Niazi et al. reported that PEG-1000 and PEG-6000 can be mixed and/or mutually dissolved by heating [40]. Therefore, we determined the melting point of a 1:1 wt.% mixture of PEG samples with molecular weights of 6000 and 1000 to control the melting temperature of PEG to the desired range.

**Figure 10:**
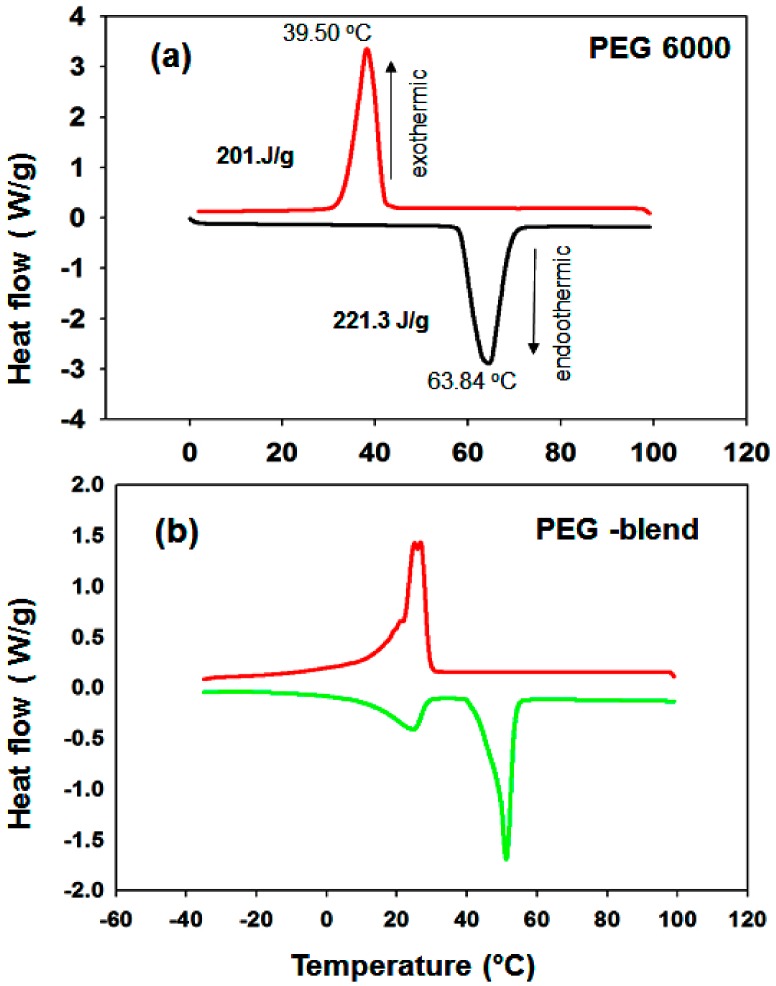
Melting–freezing DSC curves of (**a**) PEG-6000 and (**b**) PEG 1000 + 6000 PCM samples.

**Figure 11:**
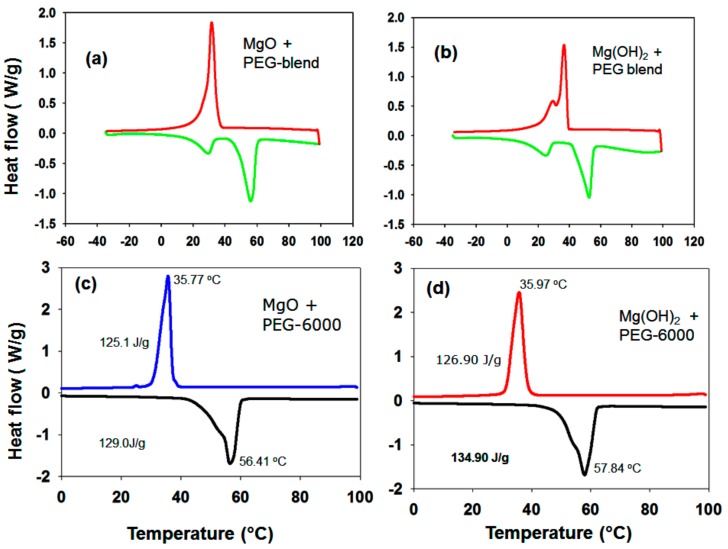
Melting–freezing DSC curves of (**a**) MgO + (PEG-1000 + 6000), (**b**) Mg(OH)_2_ + (PEG 1000 + 6000), (**c**) MgO-PEG-6000, and (**d**) Mg(OH)_2_ + PEG-6000 PCM samples.

Figure 10 shows the melting–freezing curves of (a) PEG-6000 and (b) the PEG blend. Pure PEG-6000 has a melting point of 55.30 °C and a freezing point of 28.50 °C (Figure 10a). Figure 10a shows that the latent heat of PEG is 221.3 Jg^−1^ (melting temperature T_m_ = 63.84 °C) (i.e., PEG-6000 has a high latent heat value), which is due to its linear polymer chain (CH_2_-CH_2_-O)n with hydroxyl groups on two ends. In addition, PEG crystallizes readily. In the case of the PEG blend, one sharp melting peak and also a minor broad undesired peak in the melting curve region are evident (Figure 10b). The intensity of the minor peak increases after the second and third cycles. Initially, we thought that this minor peak was due to the improper mixing of PEG-1000 and PEG-6000. However, we checked the thermal properties of PEG-1000 alone; a split peak was observed in both the melting and freezing curves. The MgO/PEG (Figure 11a) and Mg(OH)_2_/PEG blends (Figure 11b) also produce additional peaks. It has been reported that PEG-based polymeric solid–solid PCMs exhibit problematic properties during thermal energy storage if the molecular weight of the PEG is below 4000 [44]. Therefore, the remainder of the PCM samples were prepared with PEG-6000 (PEG) as the functional phase.

The melting point of the PEG/MgO sample is 56.41 °C and the freezing point is 35.77 °C (Figure 11c). The latent heat value was found to be 129 J/g during melting. A very small peak is evident in the freezing region near the sharp peak during freezing (Figure 11c). For PEG/Mg(OH)_2_, in contrast, the melting point is 57.84 °C and the freezing point is 35.97 °C. It has been reported that a narrow pore structure can reduce the melting temperature of PCMs, as is evident in our findings in the case of the Mg(OH)_2_ samples. The latent heat value is 134 J/g during melting (Figure 11d). For PEG/MgO, a value lower than that of pure PEG is obtained, probably due to the presence of the porous MgO matrix in the composite.

**Figure 12:**
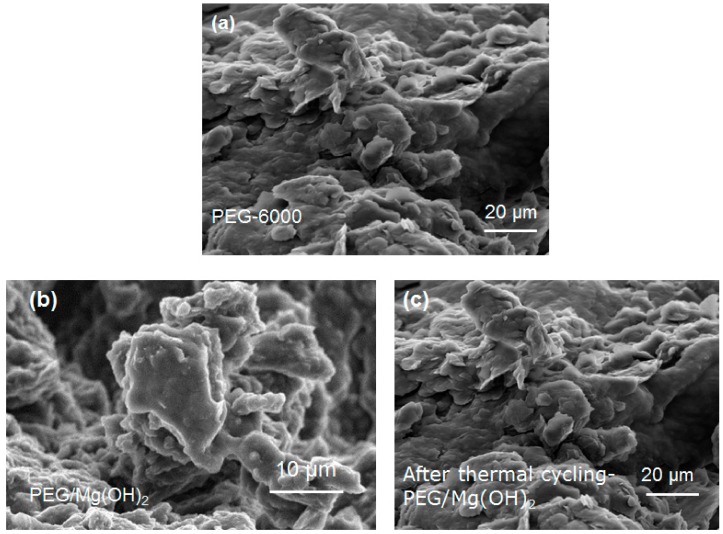
FE-SEM of (**a**) PEG-6000, (**b**) PEG/Mg(OH)_2_, and (**c**) PEG/Mg(OH)_2_ after thermal cycling.

The SEM analysis of the PEG/Mg(OH)_2_ composite shows that large amounts of PEG occupy the pores. It seems that the polymer PEG is highly dispersed on the surface of the Mg(OH)_2_ porous structure. It might happen with the help of capillary forces and the surface tension (Figure 12). We conclude that an adequate amount of PEG (0.5 g) can be encapsulated within the pores in Mg(OH)_2_ (0.2 g). The FTIR spectrum and DSC curve of the PEG/Mg(OH)_2_ PCMs are almost completely unaffected by thermal cycling, which indicates that PEG/Mg(OH)_2_ has excellent thermal reliability and reusability. After the thermal cycles, the microstructures and phase change properties of the composite were unchanged (Figure 12c).

**Figure 13:**
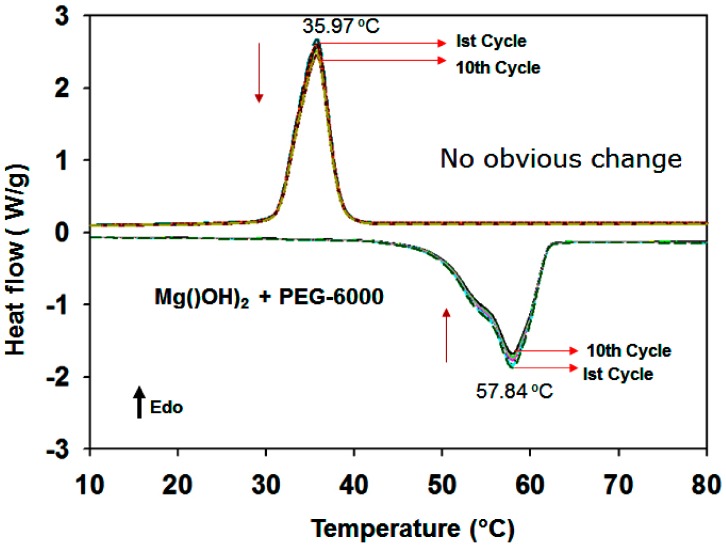
Melting–freezing DSC cycling curves of PEG/Mg(OH)_2_ PCM samples, repeated 10 times.

The thermal cycling tests show that the PEG/Mg(OH)_2_ blend provides excellent thermal reliability over at least 10 melting–solidifying cycles, which demonstrates that the Mg(OH)_2_ matrix enhances the thermal reliability of the PCM (Figure 12c and Figure 13).

Table 2 shows the thermal properties of the PCMs (e.g., the melting temperature (T_m_), the latent heat in the melting process (ΔH_m_), the freezing temperature (T_c_), and the latent heat in the freezing process (ΔH_f_)). In Table 2, it can be seen that a heat latent value of 134.90 J/g was obtained for the PEG/Mg(OH)_2_ sample, which is the highest value among the tested and reference samples. In addition, the same sample was found to have a value of supercooling that is the lowest of all the tested samples. Such results were obtained probably due to the high surface area of Mg(OH)_2_ and strong polar OH–OH bonds on the surface, which might suppress the effect of supercooling [4]. In Mg-(OH) octahedra, each Mg^2+^ cation is coordinated by 6 OH^−^ anions, while each OH^−^ anion is associated with three Mg^2+^ cations in a pyramidal geometry [40,41].

Table 2 also shows the impregnation ratio (R%), impregnation efficiency (E%), and thermal storage capability (φ%) of MgO-PEG and Mg(OH)_2_/PEG samples, which might represent the phase change performance of the prepared samples. All the parameters were calculated according to the formula (see below) of reference [22,24].

(1) $$R=\frac{\Delta H_{m},\;\mathrm{com}}{\Delta H_{m}\;\mathrm{PCM}}\times 100\%.$$

(2) $$E=\frac{\Delta H_{m},\;\mathrm{com}+\Delta H_{f},\;\mathrm{com}}{\Delta H_{m,}\;\mathrm{PCM}+\Delta H_{f},\;\mathrm{PCM}}\times 100\%.$$

(3) $$\varphi =\frac{\frac{\Delta H_{m},\;\mathrm{com}+\Delta H_{f},\;\mathrm{com}}{R}}{\Delta H_{m,}\;\mathrm{PCM}+\Delta H_{f},\;\mathrm{PCM}}\times 100\%.$$

In Equations (1)–(3), com = MgO or Mg(OH)_2_; and PCM = MgO or Mg(OH)_2_ + PEG.

The prepared Mg(OH)_2_/PEG composite achieved a high impregnation ratio of 60.96% and an efficiency of 60.00%. Additionally, the thermal storage capability was higher than 98.43%, indicating that almost all PEG molecule chains could effectively store–release heat through a phase transition. The prepared Mg(OH)_2_ is a mesoporous material that supports PEG and provides mechanical strength to the composite. As a result, the ss-CPCM retains its shape in the solid-state and does not permit seepage of the liquefied PEG. Leakage of melted PEG from the surfaces of the PCMs is prevented even after mixing PEG 71.4% with Mg(OH)_2_ 28.6% supporting material. The flower-type texture with micro porous structure of Mg(OH)_2_ is likely to enhance capturing PEG. The sample Mg(OH)_2_ showed high thermal storage enthalpy, low supercooling, and better apparent efficiency than those of MgO.

**Figure 14:**
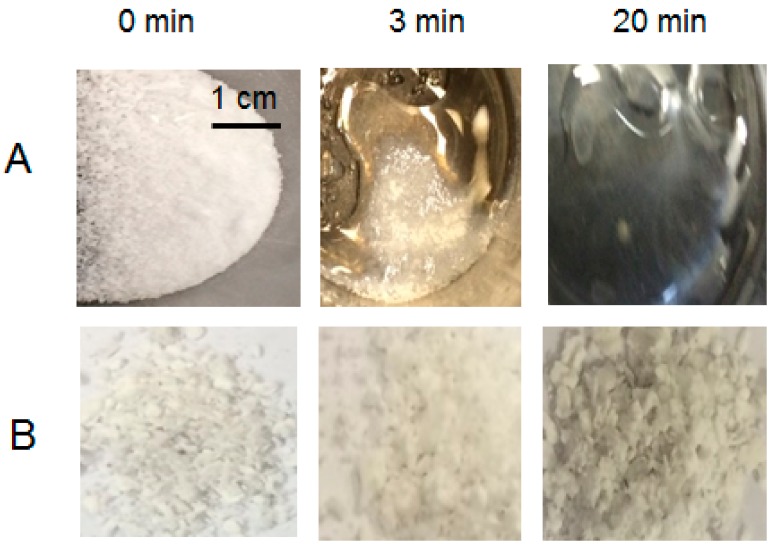
The images of heat treatment at 80 °C at different time intervals of pure PEG (**A**) and the composite PCM PEG/Mg(OH)_2_ (**B**).

The composite seepage tests of PEG/Mg(OH)_2_ were also performed by putting the melting temperature of the material slightly above the melting temperature of PEG and analyzing the stability of the structure. As shown in Figure 14, the composite of pure PEG and PEG/Mg(OH)_2_ were heated for different time phases at temperature of 80 °C. It was observed that at heat treatment of 80 °C for the duration of 8 min or above, pure PEG melted completely, while the composite of PEG/Mg(OH)_2_ retained its original solid form, as shown in Figure 14. During this phase no leakage of liquid was observed from the composite sample. This was due to the porous structure of Mg(OH)_2_, which resists leaking of molten PEG and additionally provides mechanical strength to the composite. The intake test also showed that of composite microstructure of the PEG/Mg(OH)_2_ remained unchanged during this process, as shown in Figure 14. Further, results from the SEM test presented in Figure 12c and Figure 13 shows that PEG in the matrix of Mg(OH)_2_ remains unchanged and maintains its original properties and structure.

#### 3.1.8. Thermal Conductivity

The thermal conductivity of PEG is very low [28,46], which is not desirable for practical applications. When mixed with carbon and/or inorganic materials, the conductivity of PEG is enhanced. MgO has the highest conductivity among oxide powders and Mg(OH)_2_ also showed a conductivity value of 8 Wm^−1^ K^−1^ [30]. PEG alone has a conductivity of 0.212 Wm^−1^ K^−1^, whereas the conductivity of the Mg(OH)_2_/PEG composite is 0.28 Wm^−1^ K^−1^. The results in Table 3 show that the conductivity of Mg(OH)_2_/PEG is ~58% higher than that of PEG alone. A higher conductivity is essential to enhance the heat energy storage and release or shorten the required time. In fact, a higher conductivity plays a vital role in increasing the rate of charging/discharging, which can save time and increase efficiency when these materials are applied for harnessing solar energy and recovery of waste heat. Weilong et al. found that due to pore structure of SiO_2_ a thermal conductive network was formed which enhances PEG thermal conductivity [28]. Chaoen et al. also found that the PEG/ZSM-5 thermal conductivity was increased by ZSM-5 due to thermal conductive pathway formation [25]. In this study due to use of MgO or Mg(OH)_2_ without application of any surface treatment techniques, there was no change in the thermal conductivity of composite PCMs due to decreased interfacial thermal resistance and increased PEG crystallinity for composite PCMs.

## 4. Conclusions

This study carried out the preparation and characterization of shape-stabilized PEG/MgO and PEG/Mg(OH)_2_ composites, and demonstrated the fabrication of a novel solid–liquid phase change material. The supporting materials MgO and Mg(OH)_2_ were prepared in the presence of various precipitating agents with the hydrothermal method at 200 °C for 24 h. The PEG/Mg(OH)_2_ composite was found to exhibit performance as a PCM superior to that of the PEG/MgO composite. Our comparison of the PEG/MgO and PEG/Mg(OH)_2_ systems demonstrated that Mg(OH)_2_ can be used as a supporting material for PEG-6000. Mg(OH)_2_ samples prepared with PA had a mesoporous structure with a narrow pore size distribution and the narrow pore structure is likely to play a vital role in the mitigation of supercooling effect and its high latent heat value. The PEG/Mg(OH)_2_ PCM exhibits a higher latent heat value, a higher thermal storage efficiency, and less supercooling than the PEG/MgO PCM. These properties indicate that the synthesized Mg(OH)_2_ composites are promising materials with good thermal stability for solar energy storage. Typical differential scanning calorimetry (DSC) and thermogravimetric analysis (TGA) instruments were used to confirm the results and the characteristics of the PCMs were investigated by using SEM, TEM, XPS, XRD, and FTIR spectroscopy. The PEG/Mg(OH)_2_ composite PCM might be a suitable candidate for building materials in the hot summer, whose temperature is close to 36~57 °C. A blend of PEG with MgO and Mg(OH)_2_ was also tested for suitable temperature range finding.

## Figures and Tables

**Table 1:** Textural properties of MgO and Mg(OH)_2_.

Results	Samples	
	MgO	Mg(OH)_2_
Specific surface area (m^2^g^−1^)	63.0024	184.9750
Pore volume (ccg^−1^)	0.5519	0.8421
Mean pore diameter (nm)	20.2913	10.5147

**Table 2:** DSC results of pure PEG-6000 (PEG) and PEG/MgO and PEG/Mg(OH)_2_ composite PCMs and comparison with that of different PEG composite PCMs in literature.

Sample	T_f_ (°C)	T_m_ (°C)	ΔH_f_ (J/g)	ΔH_m_ (J/g)	ΔT_s_	R (%)	E (%)	φ (%)
* PEG (6000)	39.5	63.84	201.0	221.3	24.34	-		
** PEG-6000/MgO	35.77	56.41	110.4	129.0	20.64	58.29	56.69	97.25
*** PEG-6000/Mg(OH)_2_	37.97	57.84	118.5	134.90	19.89	60.96	60.00	98.43
^1^ PEG1000/MgO	18.30	34.40	-	61.62	16.10		64.6	
^2^ PEG-10,000/SiO_2_	-	61.61	-	162.9	-	-		
^3^ PEG-1000/SiO_2_-ß-AIN	45.13	60.41	161.4	132.9	15.28	-		
^4^ PEG/CaO_4_Si-6000	44.19	57.59	99.53	113.60	13.40	-		

(*, ** and ***) Represents the present study; (-) = Data are not available, (^1^) = Reference [23], (^2^) = Reference [28], (^3^) = Reference [45], (^4^) = Reference [22].

**Table 3:** Thermal conductivity of the pure PEG-6000 and PEG-6000/MgO and PEG-6000/Mg(OH)_2_ composites *.

Sample	Conductivity (Wm^−1^K^−1^)
PEG-6000	0.2124
PEG-6000/MgO	0.7423
PEG-6000/Mg(OH)_2_	0.3334

* = Reference [25].
